# Isolation of Foreign Material-Free Endothelial Progenitor Cells Using CD31 Aptamer and Therapeutic Application for Ischemic Injury

**DOI:** 10.1371/journal.pone.0131785

**Published:** 2015-07-06

**Authors:** Jung Won Yoon, Il Ho Jang, Soon Chul Heo, Yang Woo Kwon, Eun Jung Choi, Kwang-Hee Bae, Dong-Soo Suh, Seung-Chul Kim, Seungmin Han, Seungjoo Haam, Jongha Jung, Kiseok Kim, Sung Ho Ryu, Jae Ho Kim

**Affiliations:** 1 Department of Physiology, School of Medicine, Pusan National University, Yangsan 626–870, Republic of Korea; 2 Functional Genomics Research Center, Korea Research Institute of Bioscience and Biotechnology, Daejeon, Republic of Korea; 3 Departments of Obstetrics and Gynecology, School of Medicine, Pusan National University, Yangsan 626–870, Republic of Korea; 4 Department of Chemical and Biomolecular Engineering, College of Engineering, Yonsei University, Seoul 120–749, Republic of Korea; 5 Aptamer Sciences Inc. Postech Biotech Center, San 31 Hyoja-Dong, Pohang 790–784, South Korea; 6 Department of Life Sciences, Pohang University of Science and Technology, Pohang 790–784, South Korea; 7 Research Institute of Convergence Biomedical Science and Technology, Pusan National University Yangsan Hospital, Yangsan 626–770, Gyeongsangnam-do, Republic of Korea; Wake Forest Institute for Regenerative Medicine, UNITED STATES

## Abstract

Endothelial progenitor cells (EPCs) can be isolated from human bone marrow or peripheral blood and reportedly contribute to neovascularization. Aptamers are 40-120-mer nucleotides that bind to a specific target molecule, as antibodies do. To utilize apatmers for isolation of EPCs, in the present study, we successfully generated aptamers that recognize human CD31, an endothelial cell marker. CD31 aptamers bound to human umbilical cord blood-derived EPCs and showed specific interaction with human CD31, but not with mouse CD31. However, CD31 aptamers showed non-specific interaction with CD31-negative 293FT cells and addition of polyanionic competitor dextran sulfate eliminated non-specific interaction without affecting cell viability. From the mixture of EPCs and 293FT cells, CD31 aptamers successfully isolated EPCs with 97.6% purity and 94.2% yield, comparable to those from antibody isolation. In addition, isolated EPCs were decoupled from CD31 aptamers with a brief treatment of high concentration dextran sulfate. EPCs isolated with CD31 aptamers and subsequently decoupled from CD31 aptamers were functional and enhanced the restoration of blood flow when transplanted into a murine hindlimb ischemia model. In this study, we demonstrated isolation of foreign material-free EPCs, which can be utilized as a universal protocol in preparation of cells for therapeutic transplantation.

## Introduction

Nucleic acid aptamers are single-stranded oligonucleotides, typically 40-120-mers, and bind to a specific target with high affinity, as antibodies do [1]. Aptamers can be screened from oligonucleotide libraries by systematic evolution of ligands by exponential enrichment (SELEX) [2]. Aptamers have attracted attention in the field of clinical diagnosis and therapy because of the several advantages over antibodies, including low immunogenicity, efficient entry into biological compartments due to smaller size, bacterial contamination-free production, stability in storage, easy and rapid production, and conjugation chemistries for attachment of dyes or functional groups during synthesis [3]. The first aptamer drug was approved by the US Food and Drug Administration in 2005, and many others are in clinical pipelines [4, 5].

Endothelial progenitor cells (EPCs) incorporate into foci of physiological or pathological postnatal neovascularization [6]. EPCs were first isolated from adult peripheral blood and later shown to derive from bone marrow and other tissues [7]. EPCs contribute to vascular regeneration by direct incorporation into newly forming blood vessels or by secretion of pro-angiogenic factors [8, 9]. The widely used EPC culture starts with peripheral blood- or bone marrow-derived mononuclear cells in endothelial growth factor-supplemented media. The adherent cells in culture exhibit certain endothelial characteristics, such as expression of endothelial lineage markers, including CD31, migration toward angiogenic growth factor gradient, formation of tube-like structures, and contribution to repair of ischemic tissues after *in vivo* transplantation [10–13]. Transplanting EPCs is expected to provide a novel therapeutic opportunity for treatment of ischemic disease through functional contribution to formation of new vasculature, and various clinical trials are now ongoing [6, 14, 15].

CD31, also known as PECAM-1, is a cell adhesion and signaling receptor highly expressed in endothelial cells and to various degrees on several non-erythroid hematopoietic cells [16]. CD31 is a member of the Ig-superfamily and a type I transmembrane glycoprotein with six extracellular Ig-like homology domains [17]. The major ligand for CD31 is CD31, a homophilic interaction mediated by Ig-like domain 1 [18]. CD31 plays a role in mediating homotypic adhesions between neighboring endothelial cells and adhesions of leukocytes on endothelial cells during transendothelial migration [19, 20]. Recent studies have demonstrated that EPCs could be isolated from mouse and human bone marrow and human peripheral blood with CD31 antibodies [21, 22]. Isolated CD31-positive cells exhibited robust angiogenic and vasculogenic activity *in vitro* and promoted vascular repair in a hindlimb ischemia mouse model when transplanted. These results demonstrated that CD31 is an excellent marker in isolation of angiogenic and vasculogenic cells for therapeutic cell transplantation [23, 24].

For clinical application of EPCs, human cord blood can be used for isolation of EPCs; however, cord blood contained not only EPCs but also various other cell types, such as immune cells and mesenchymal stem cells. Therefore, isolation of EPCs from other cell types within cord blood could increase therapeutic efficacy and safety in treatment of ischemic diseases. For isolation of clinically applicable EPCs, magnetic cell sorting using antibody-conjugated microbeads is currently used [6, 25]. However, there is some limitation in clinical application of the antibody-conjugated microbeads in patients with hypersensitivity to murine proteins or iron dextran due to immunogenic problem of mouse antibodies and toxicity of iron dextran microbeads [26, 27]. Therefore, it is necessary to develop new cell isolation methods for preparation of foreign material-free EPCs.

In this study, we identified three clones of CD31 aptamer that bound to EPCs expressing CD31 on the surface. CD31 aptamers specifically recognized human CD31, and we established a protocol for isolation of EPCs from cell mixture using CD31 aptamers with purity and efficiency comparable to those of antibody isolation. In addition, we efficiently decoupled CD31 aptamers from isolated EPCs, which is a step forward in preparation of cells for therapeutic transplantation. EPCs isolated from cultured cord blood mononuclear cells (MNCs) with CD31 aptamers improved blood flow and vascular function when transplanted into ischemic mouse model. These results demonstrate a successful identification and application of CD31 aptamers for isolating functional EPCs in therapeutic application.

## Materials and Methods

### Materials

Trypsin-EDTA and Hank's Balanced Salt Solution (HBSS) were purchased from Invitrogen (Carlsbad, CA). Dulbecco’s Modified Eagle medium (DMEM; LM001-05) and 1× phosphate buffered saline (pH = 7.4) (LB001-02) were purchased from Welgene (Daegu, South Korea). FITC-CD31 antibody (555445), isotype cocktail-C (558659), 7-Aminoactinomycin D (7-AAD) (51-68981-E), and streptavidin magnetic beads (11205D) were purchased from BD Bioscience (San Jose, CA). LS columns (130-042-401) and anti-FITC microbeads (130-048-701) were purchased from Miltenyi Biotech (Bergisch Gladbach, Germany). CD31-Cy5 aptamers, CD31-biotin aptamers, and scrambled epidermal growth factor receptor (EGFR)-FITC aptamers were purchased from Aptamer Science Inc. (Pohang, South Korea). Dextran sulfate (DxSO_4_) was purchased from American International Chemial Inc. (9011-18-1, Framingham, MA). Endothelial cell basal medium-2 (CC-3156) and EGM-2MV BulletKit (CC-4147) were purchased from Lonza Ltd (Basel, Switzerland). 293FT cells were purchased from American Type Culture Collection. Fetal bovine serum was purchased from Gibco (Life Technologies) and penicillin-streptomycin solution was from HyClone (GE Healthcare, Uppsala, Sweden). Rhodamine-labeled lectin (Ulex europaeus agglutinin I, RL-1062) and mounting medium with DAPI (H-1200) were purchased from Vector Laboratories (Burlingame, CA). Gelatin (G1303), MgCl_2_ (M2670), BSA (A7946), and all other chemicals were purchased from Sigma-Aldrich (St. Louis, MO).

### Cell Culture

Human EPCs were isolated from human umbilical cord blood and collected in disposable sterile pyrogen-free bags containing anticoagulant (Green Cross, Seoul, Korea). Written informed-consent was obtained from all donors and the protocol was approved by the Institutional Review Board of Pusan National University Hospital (Permit Number: H-1302-005-015). MNCs were isolated from blood with Ficoll-Paque PLUS (17-1440-02, GE healthcare) as described previously [13]. Cells were seeded on culture dishes coated with 0.1% gelatin and maintained in EGM-2MV BulletKit. The medium was exchanged in 24 hours after the initial plating for removal of non-adherent cells and was exchanged every day for the first week. Colonies of EPCs appeared 7–10 days after the initial isolation. Non-adherent cells were removed, and the adherent cells were trypsinized and replated at a density of 1×10^6^ cells per well. Expression of EPC-specific cell surface markers, including CD34, CD31, CD133, c-kit, Flk1, CXCR4, and CD144, was confirmed by flow cytometry analysis. Hematopoietic lineage markers such as CD11b, CD14, and CD45 were not expressed in EPCs, supporting the commitment of EPCs to the endothelial lineage. 293FT cells were cultured in high glucose (4.5 g/L) DMEM supplemented with 10% fetal bovine serum and 100 U/mL penicillin-streptomycin. All cells were maintained at 37°C in a 5% CO_2_ atmosphere.

### Flow Cytometry Analysis

Aptamer stocks were prepared by dissolving dry pellet to be 20 μM with sterile distilled water and stored at -20°C. Aptamers were heated at 95°C for 5 minutes and cooled down at room temperature for formation of the proper conformation before application. Fluorescent dye-labeled aptamers were incubated with cells at room temperature for 15 minutes in 100 μL of aptamer binding buffer (1× PBS containing 2% FBS, 5 mM MgCl_2_, 0.2mM DxSO4). The incubated cells were washed once with 1 mL of aptamer binding buffer before further analysis. Cells labeled with aptamers or antibodies were analyzed by BD FACSCanto II (BD Biosciences) by counting 10,000 events. 7-AAD was added at a 1:100 dilution to distinguish live cells from dead cells. Obtained fluorescence signals were analyzed using the FACSDiva (ver 6.1.3, BD Biosciences) or the FlowJo (ver 10, Tree Star Inc.).

### Differentiation and Isolation of CD31-positive Cells from Mouse Embryonic Stem Cells

Ainv15 mouse embryonic stem cells (ESCs) were cultured on mitomycin-C-treated mouse embryonic fibroblasts in ESC culture media consisting of DMEM supplemented with 1× non-essential amino acids, 1× GlutaMAX (35050–061, Life Technologies), 15% FBS, 1× penicillin-streptomycin, 0.1 mM β-mercaptoethanol, and 1000 U/mL leukemia inhibitory factor (ESG1106, Merck Millipore). Mouse embryonic fibroblasts were prepared as described previously [28] and maintained in mouse embryonic fibroblast culture medium (DMEM with high glucose, 1× GlutaMAX, 10% FBS, and 1× penicillin-streptomycin). For hanging-drop culture of EBs, dissociated ESCs were spotted on a petri-dish lid with 20 μL drops of cells (50,000 cells/mL) in ES cell medium without leukemia inhibitory factor. Embryonic bodies were harvested two days after hanging-drop formation, transferred to a petridish, and maintained on the shaker in a humidified incubator at 37°C under 5% CO_2_ with change of media on day 4. EBs were harvested on day 6 and subjected to analysis by flow cytometry.

### Magnetic Cell Sorting of EPCs

The magnetic cell sorter (Miltenyi Biotec) was used for sorting cells with magnetic beads. Cells were incubated with biotin-conjugated CD31 aptamers at room temperature or FITC-labeled CD31 antibodies on ice in 100 μL of aptamer binding buffer for 15 minutes. Cells were washed with 1 mL of aptamer binding buffer three times and incubated with streptavidin-magnetic beads (120-000-287, Miltenyi Biotec) or anti-FITC magnetic beads (120-000-293, Miltenyi Biotec) in 100 μL of aptamer binding buffer on ice for 20 minutes. After incubation, cells were washed with 1 mL of aptamer binding buffer once and transferred to LS columns following the company protocol. Briefly, LS column was placed in the magnet and pre-hydrated with 1 mL of aptamer binding buffer. Cell-aptamer or antibody-magnetic bead complexes were resuspended in 500 mL aptamer binding buffer and applied to LS column, followed by washing with 1 mL aptamer binding buffer three times. LS column was removed from the magnet and cell-aptamer or antibody-magnetic bead complexes were eluted with 1 mL of aptamer binding buffer and plunger.

### Decoupling of EPCs from CD31 Aptamer-EPC Complexes

EPCs bound with CD31-biotin aptamers and streptavidin magnetic-beads were incubated in decoupling buffer (1× PBS containing 2% FBS, 5 mM MgCl_2_, 5 mM DxSO_4_) at 4°C for 15 minutes. After incubation, cells were applied to a magnetic cell sorter column pre-hydrated with 3 mL decoupling buffer. Pass-through fraction was collected to harvest decoupled EPCs, and residual cells in the column were collected as un-decoupled fraction. The decoupled EPCs were centrifuged and washed with HBSS three times, and cells were then subjected to flow cytometry analysis with CD31 antibodies for assessment of purity, and cell numbers were counted for assessment of yield.

### Cell Imaging

Cells on 0.1% gelatin coated cover slip were fixed with 4% paraformaldehyde in 1×PBS followed by incubation in PBS containing 5% BSA and 0.5% triton X-100 (2052–6356, SHOWA, Tokyo, Japan) at room temperature for 1 hour to limit non-specific binding. These specimens were incubated with anti-CD31 antibodies (1:500 dilution) (ab28364, Abcam, Cambridge, UK) or rhodamine-labeled lectin at 4°C overnight. After washing three times with PBS for 5 minute individual incubation, CD31 antibody-incubated samples were further incubated with Alexa Fluor 488-conjugated anti-rabbit antibody (1:200 dilution) (A-11008, Life Technologies) at room temperature for 1 hour. For confocal imaging with aptamers, cells were co-incubated with the biotin-labeled CD31 aptamers and Alexa Flour 488-conjugated streptavidin (S11223, Life Technologies) at 37°C for 1 hour, fixed with paraformaldehyde, and washed three times. The specimens were mounted in mounting medium with DAPI for visualization of nuclei. For DiI-Ac-LDL labeling, cells on 0.1% gelatin coated cover slip in culture media were incubated with DiI-Ac-LDL (1:20 dilution, BT902, Biochemical Technology, USA) for 4 hours at 37°C. The specimens were fixed with 4% paraformaldehyde and finally washed with 1× PBS and mounted with Vectashield medium (Vector Laboratories) containing 4',6-diamidino-2-phenylindole (DAPI). Confocal microscope system (Olympus FluoView FV1000, Olympus Corp., Tokyo, Japan) was used for collection of images.

### Mouse Hindlimb Ischemia Model and Blood Flow Measurement

This study was carried out in strict accordance with the recommendations in the Guide for the Care and Use of Laboratory Animals of the National Institutes of Health. The protocol was approved by the Pusan National University Institutional Animal Use and Card Committee. (Permit Number: PNU-2009-0033). All surgery was performed under sodium pentobarbital anesthesia, and all efforts were made to minimize suffering. BALB/CA-nu/nu (male, age 8 weeks, weighing 18–20 g) were anesthetized with an intraperitoneal injection of 400 mg/kg 2,2,2-tribromoethanol for operative resection of one femoral artery. The femoral artery was excised from its proximal origin as a branch of the external iliac artery to the distal point where it bifurcates into the saphenous and popliteal arteries. After arterial ligation, HBSS or EPCs (1×10^6^ in 60 μl HBSS) were injected intramuscularly into three sites near ischemic injury (20 μL/each site) with Hemilton syringe (1710, Reno Nevada, USA). The connective tissues of the subcutis were closed with interrupted 6–0 polypropylene suture and the skin closed with wound clips or 6–0 polypropylene suture. Post-surgery animals received subcutaneous injection of buprenorphine (0.03 mg/kg twice daily) for pain relief over 2 days, and meloxicam solution (0.001 mg/g) was administered in the water for up to ten days post operatively to minimize any pain as a result of surgery. The health status of the mice was monitored once a day for four weeks by observing changes in breathing pattern, overt pain or distress, locomotion difficulties, proper food and water intake, and skin color. All mice used for data collection did not show severe locomotion difficulties and consumed food and water properly. The extent of necrosis was evaluated according to remained muscle and skin in mouse hindlimb. The scores of necrosis were judged as follows; no necrosis on ischemic hindlimb is grade 0, grade I is limited necrosis of toe, grade II is necrosis progressed up to foot, grade III is up to ankle, grade IV is up to knee. Blood flow of the ischemic and normal limb was measured using a laser Doppler perfusion imaging (LDPI) analyzer (Moor instruments, Devon, UK) on days 0, 7, 14, 21, and 28 after induction of hindlimb ischemia. Perfusion of the ischemic and non-ischemic limb was calculated on the basis of colored histogram pixels. Red and blue colors indicate high and low perfusion, respectively. Blood perfusion is expressed as the LDPI index representing the ratio of ischemic versus non-ischemic limb blood flow. A ratio of 1 before surgery indicates equal blood perfusion of both legs. Mice were sacrificed by euthanizing with carbon dioxide followed by cervical dislocation on day 28 after surgery.

### Immunostaining Analysis of Ischemic Limbs

For immunostaining of the tissue specimens, hindlimb muscles were removed and fixed with acetone, methyl benzoate, xylene, and paraffin-embedded. Sections (6 μm in thickness) were taken from the paraffin-embedded specimens at 150 μm intervals, stained with rat anti-CD31 antibody (H553373, BD Pharmingen). The specimens were incubated with Alexa Flour 488 goat anti-rat antibody (A-11006, Life Technologies), followed by washing and mounting in Vectashield medium containing DAPI for visualization of nuclei. The stained sections were visualized using laser scanning confocal microscopy (Olympus FluoView FV1000). Capillary densities were assessed by counting the number of CD31-positive features per high power field (×400). Three randomly-selected microscopic fields form three serial sections in each tissue block were examined for capillary counting.

## Results

### Identification of CD31-specific Aptamers

**Fig 1:**
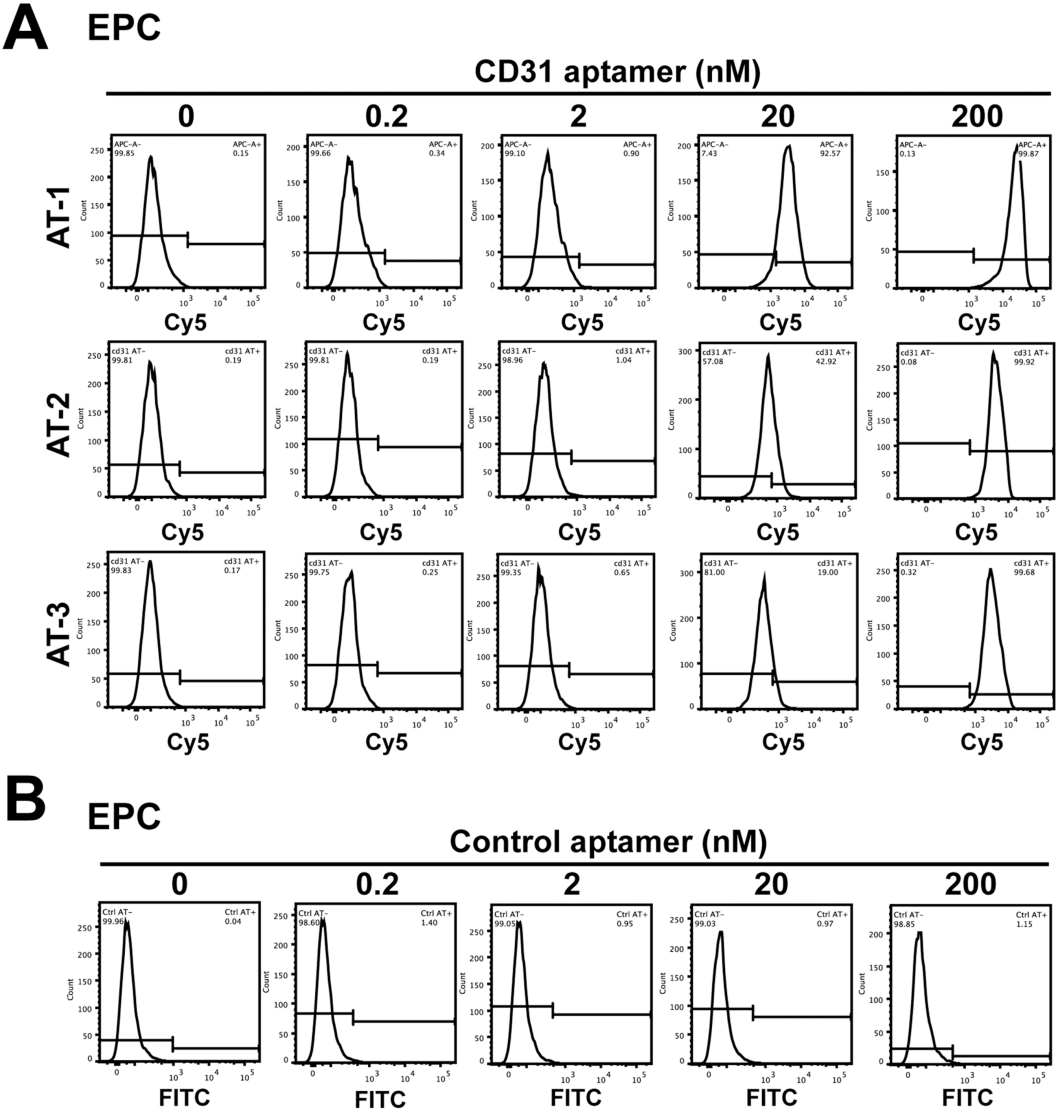
CD31 aptamers interact with human EPCs. (A) Flow cytometry analysis of EPCs after individual incubation with various concentrations (0, 0.2, 2, 20, and 200 nM) of three CD31 aptamer clones (AT-1, AT-2, and AT-3, Cy5-labeled) is shown. (B) Flow cytometry analysis of EPCs after incubation with various concentrations (0, 0.2, 2, 20, and 200 nM) of control aptamers (FITC-labeled) is shown (n = 5).

In targeting distinct cells with functional cargo in complex mixtures of cells, aptamers can provide advantages over antibodies, which are most widely used in scientific and clinical applications, in the short generation time due to the entire chemical process and the easiness of modification with functional groups [29]. Aptamers are generated through an *in vitro* process termed SELEX, and a conservative estimate of the success rate is ~50% [30]. In addition, application of *in vitro* selected aptamers to cells requires fine calibration. Among CD31 aptamer clones selected using the purified extracellular domain of CD31, we tested three clones that showed the highest affinity in the application of recognizing CD31 on the cell surface. EPCs showed high expression of CD31 in analysis by flow cytometry using CD31 antibodies (S1 Fig). In application of CD31 aptamers to EPCs at various concentrations (0.2, 2, 20, and 200 nM), clones 1, 2, and 3 (AT-1, AT-2, and AT-3), each of which was labeled with Cy5, showed interaction with EPCs (Fig 1A). The intensity of the signal from CD31 aptamers continued to increase from 0.2 to 200 nM, and 400 nM treatment did not further increase the signal intensity (Fig 1A, data not shown). Among them, clone 1 (AT-1) showed the strongest signal, and we proceeded with the rest of the experiments using CD31 aptamer clone 1, if not stated otherwise (S1 Table). Incubation of ECPs with control scrambled EGFR-FITC aptamers did not generate a positive signal (Fig 1B). These results suggest that we generated aptamers that recognize CD31 on the surface of EPCs.

### Resolving non-specific interaction of CD31 aptamers with dextran sulfate

**Fig 2:**
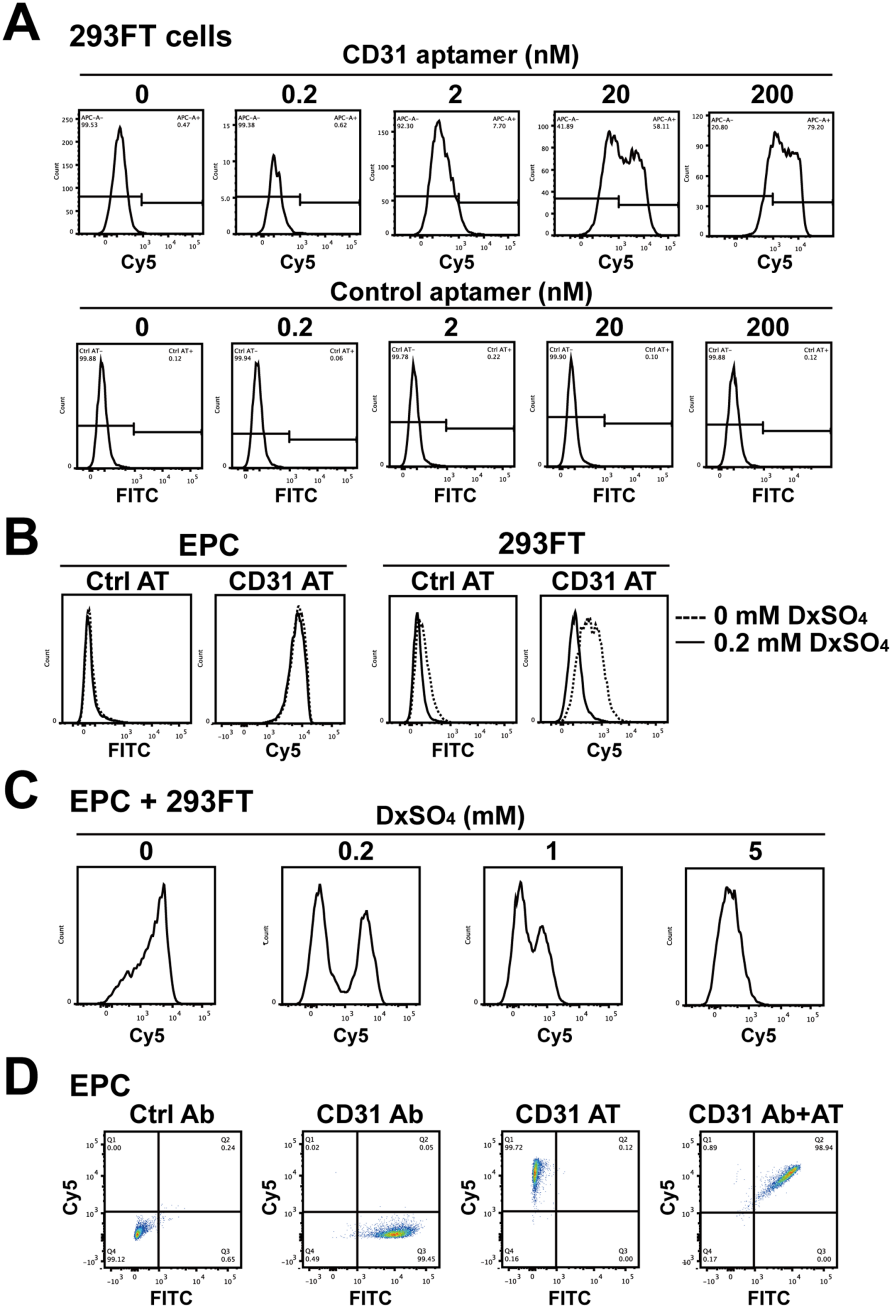
Dextran sulfate effectively reduces the non-specific interaction of CD31 aptamers. (A) 293FT cells were incubated with various concentrations (0, 0.2, 2, 20, and 200 nM) of CD31 aptamer clone 1 (AT-1, Cy5-labeled) or control aptamers (FITC-labeled) and subjected to flow cytometry analysis (n = 5). (B) EPCs or 293FT cells were separately incubated with control aptamers (Ctrl AT, FITC-labeled) or CD31 aptamers (AT-1, Cy5-labeled, 200 nM) with or without 0.2 mM dextran sulfate and subjected to flow cytometry. The overlap of histograms from 0 and 0.2 mM dextran sulfate experiments is shown (n = 5). (C) The mixture of EPCs and 293FT cells was incubated with CD31 aptamers (AT-1, Cy5-labeled, 200 nM) and various concentrations (0, 0.2, 1, and 5 mM) of dextran sulfate, followed by flow cytometry analysis (n = 3). (D) EPCs were incubated with CD31 antibodies (FITC-labeled) alone, CD31 aptamers alone (AT-1, Cy5-labeled, 200 nM), or with both CD31 antibodies and CD31 aptamers and subjected to flow cytometry analysis. Two-dimensional plots are shown (n = 5).

Although CD31 aptamers showed strong interaction with EPCs, the specificity of the interaction was not confirmed. In flow cytometry analysis with CD31 antibodies, 293FT cells did not show expression of CD31 (S1 Fig). When 293FT cells were incubated with CD31 aptamer clone 1 at various concentrations from 0.2 to 200 nM, CD31 aptamers showed non-specific interaction with 293FT cells whereas control aptamers did not show interaction (Fig 2A). To resolve the non-specific interaction of CD31 aptamers, we introduced dextran sulfate, which is used for controlling hybridization temperature and blocking non-specific interaction in polymerase chain reaction, as an anionic competitor to incubation of CD31 aptamers with EPCs or 293FT cells [31, 32]. Without dextran sulfate, CD31 aptamers showed interaction with both EPCs and 293FT cells. Addition of 0.2 mM dextran sulfate did not inhibit the specific interaction of CD31 aptamers with EPCs. However, the interaction of CD31 aptamers with 293FT cells was completely abolished to the level of the control aptamers (Fig 2B). Incubation of CD31 aptamers with the mixture of EPCs and 293FT cells without dextran sulfate did not distinguish two populations (Fig 2C). However, addition of 0.2 mM dextran sulfate to the mixture of EPCs and 293FT cells clearly separated two peaks, indicating resolution of the non-specific interaction. Higher concentrations of dextran sulfate, such as 1 and 5 mM, inhibited the specific interaction of CD31 aptamers, suggesting that 0.2 mM dextran sulfate is the optimal concentration for blocking the non-specific interaction but allowing the specific interaction. The same patterns were observed with different clones of CD31 aptamers (S2 Fig). Single- or double-staining of EPCs with CD31 antibodies and/or CD31 aptamers in the presence of 0.2 mM dextran sulfate showed that CD31 aptamers are as efficient as CD31 antibodies in detecting EPCs (Fig 2D). These results demonstrate that 0.2 mM dextran sulfate resolves the non-specific interaction of CD31 aptamers and is required for application of CD31 aptamers in recognition of EPCs.

Dextran, which is used in intravenous fluids, functions as a volume expander and in parenteral nutrition. Dextran, however, can apply osmotic pressure, and a certain molecular weight of dextran sulfate sodium causes colitis in mice when added to drinking water [33]. Thus, we tested whether dextran sulfate could have a deleterious effect on cell viability. When EPCs or 293FT cells were incubated with various concentrations (0, 0.2, 0.5, 1, or 2 mM) of dextran sulfate, neither EPCs nor 293FT cells showed differences in 7-AAD positivity when compared with no treatment control (S3 Fig). To further confirm the effect of dextran sulfate on cell viability, EPCs or 293FT cells were subjected to MTT assay. As shown in S3 Fig, incubation of EPCs or 293FT cells with various concentrations (0, 0.2, 0.5, 1, or 2 mM) of dextran sulfate did not change metabolic activity of cells. These results indicate that incubation of EPCs or 293FT cells with 0.2 mM dextran sulfate for blocking the non-specific interaction of CD31 aptamers does not affect cell viability.

To test species specificity of CD31 aptamers, we compared the interaction of CD31 aptamers with human EPCs and CD31-positive mouse cells, which was prepared from differentiation of mouse ESCs into embryoid bodies. As shown in S4 Fig, day 6 mouse embryoid bodies generated CD31-positive cells recognized by anti-mouse CD31 antibodies. However, neither CD31 aptamers nor anti-human CD31 antibodies showed the interaction with CD31-positive cells derived from mouse embryoid bodies. Taken together, these results demonstrate that we generated CD31 aptamers specific to human CD31 and established a protocol for recognizing EPCs from cell mixture without decreasing cell viability.

### Visualizing EPCs using CD31 aptamers

**Fig 3:**
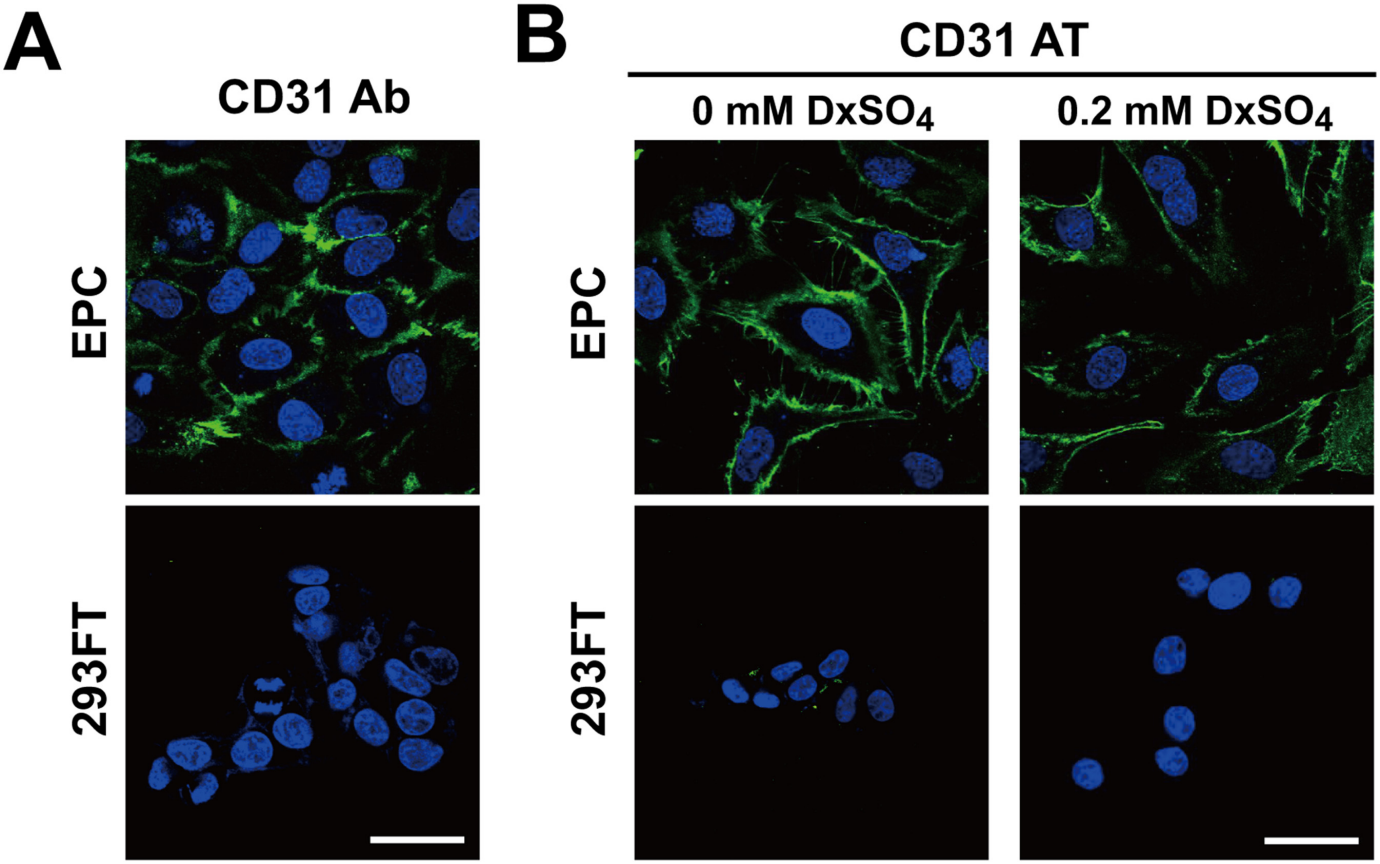
CD31 aptamers specifically stain EPCs for visualization with fluorescence microscopy. (A) EPCs or 293FT cells were stained with CD31 antibodies (Alexa Fluor 488-labeled) and DAPI (blue). Images were taken by confocal microscope (Olympus FluoView FV1000). (B) EPCs or 293FT cells were stained with CD31 aptamers (AT-1, biotin-labeled, 400 nM) at 37°C for 1 h with or without 0.2 mM dextran sulfate, followed by staining with streptavidin-Alexa Fluor 488 and DAPI (blue). Images were taken by confocal microscope (Olympus FluoView FV1000). Scale bar = 40 μm (n = 3).

We tested whether CD31 aptamers could be used in visualizing EPCs as antibodies in immunocytochemistry. When EPCs or 293FT cells were plated separately, CD31 antibodies specifically stained EPCs (Fig 3A). Following the immunocytochemistry protocol with aptamer binding buffer, CD31 aptamers were applied to EPCs or 293FT cells. Without dextran sulfate, CD31 aptamers showed strong interaction with EPCs (Fig 3B). Low level interaction with 293FT cells was also observed though low level interaction is weaker than that in flow cytometry analysis probably due to increased washing in immunocytochemistry protocol. However, addition of 0.2 mM dextran sulfate eliminated non-specific interaction of CD31 aptamers with 293FT cells but conserved the specific interaction with EPCs. These results demonstrate that CD31 aptamers can be used for visualizing EPCs with efficiency comparable to that of CD31 antibodies in immunocytochemistry.

### Isolating EPCs using CD31 aptamers

**Fig 4:**
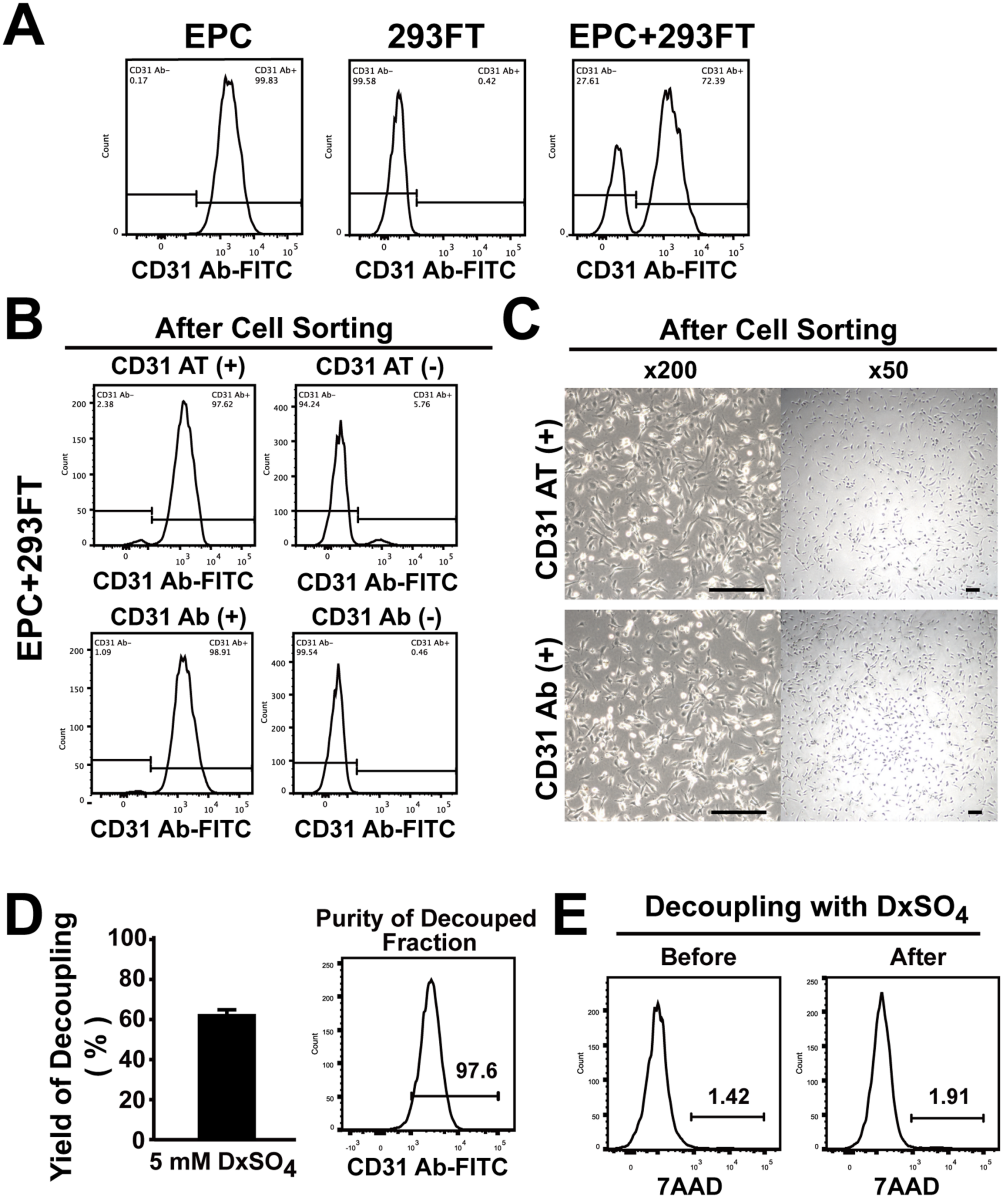
CD31 aptamers isolate EPCs from the mixture of EPCs and 293FT cells and EPCs are decoupled from CD31 aptamers. (A) EPCs, 293FT cells, or the mixture of EPCs and 293FT cells were analyzed by flow cytometry after staining cells with FITC-labeled anti-human CD31 antibodies (n = 3). (B) The mixture of EPCs and 293FT cells was subjected to magnetic bead sorting using CD31 aptamers (AT-1, biotin-labeled) (upper panel) or CD31 antibodies (lower panel). (+) indicates the fraction with positive selection and (-) indicates the fraction with negative selection. Flow cytometry analysis of each fraction with CD31 antibodies (FITC-labeled) after isolation is shown (n = 3). (C) Bright field images of cultured cells in (+) fraction after magnetic bead isolation of the mixture of EPCs and 293FT cells with CD31 aptamers (upper panel) or CD31 antibodies (lower panel) are shown. Scale bar = 200 μm (n = 3). (D) Decoupling of EPCs from CD31 aptamer-EPC complexes by treatment with DxSO_4_. EPCs were decoupled from the CD31 aptamer-EPC complexes and recovery yield was calculated by comparing cell numbers before and after decoupling with DxSO_4_ (left panel) (n = 4). Flow cytometry analysis of decoupled cells with CD31 antibody is shown in the right panel. (E) Viability of EPCs before and after decoupling with DxSO_4_ is shown by flow cytometry analysis (n = 5).

EPCs have been shown to provide therapeutic benefits when transplanted into ischemic injury models [12, 34]. We tested whether CD31 aptamers could isolate EPCs from the mixture of cells. We prepared, the cell mixture comprised of ~72% EPCs and ~28% 293FT cells (Fig 4A). To test the efficiency of cell sorting with CD31 aptamers, the mixture of EPCs and 293FT cells was subjected to magnetic bead sorting using biotin-labeled CD31 aptamers and streptavidin magnetic beads. The mixture of EPCs and 293FT cells was also subjected to magnetic bead sorting using CD31 antibodies for comparison of the sorting efficiency. In analysis of post-sort fractions using CD31 antibodies, the EPC fraction isolated by CD31 antibodies showed 98.9% purity and 99.5% yield. The EPC fraction isolated by CD31 aptamers showed 97.6% purity and 94.2% yield, comparable to those of isolation by CD31 antibodies (Fig 4B). When isolated EPCs were subjected to culture, both EPC fractions from CD31 aptamer-isolation and CD31 antibody-isolation showed proliferating EPCs, whose morphology is distinct from that of 293FT cells (Fig 4C). These results demonstrate that CD31 aptamers can isolate EPCs with high purity and high yield comparable to those of CD31 antibodies from the mixture of cells.

### Decoupling of CD31 aptamers from EPCs

The presence of foreign materials on the surface of cells intended for transplantation for therapeutic purposes is a cause for concern regarding decrease of efficacy related to immune reactions. We tested decoupling of CD31 aptamers from EPCs after binding of CD31 aptmer to EPCs. After incubation of EPCs with CD31 aptamers in the presence of 0.2 mM dextran sulfate, EPCs were subjected to magnetic bead isolation protocol to emulate purification process without contaminating 293FT cells. EPCs were eluted from magnetic column and subsequently subjected to a decoupling process in order to release EPCs from aptamers and magnetic beads. Incubation of EPC-CD31 aptamer-magnetic bead complexes with decoupling buffer (aptamer binding buffer containing 5 mM dextran sulfate) at 4°C for 15 minutes and passaging through magnetic column showed .decoupling of EPCs from aptamer-magnetic bead complexes with 63.8% yield (Fig 4D). Decoupled EPCs maintained CD31 expression on the surface and did not show a change in viability compared with that prior to decoupling (Fig 4D and 4E). These results demonstrate that foreign material-free EPCs can be harvested from the isolation process using CD31 aptamers and magnetic beads.

**Fig 5:**
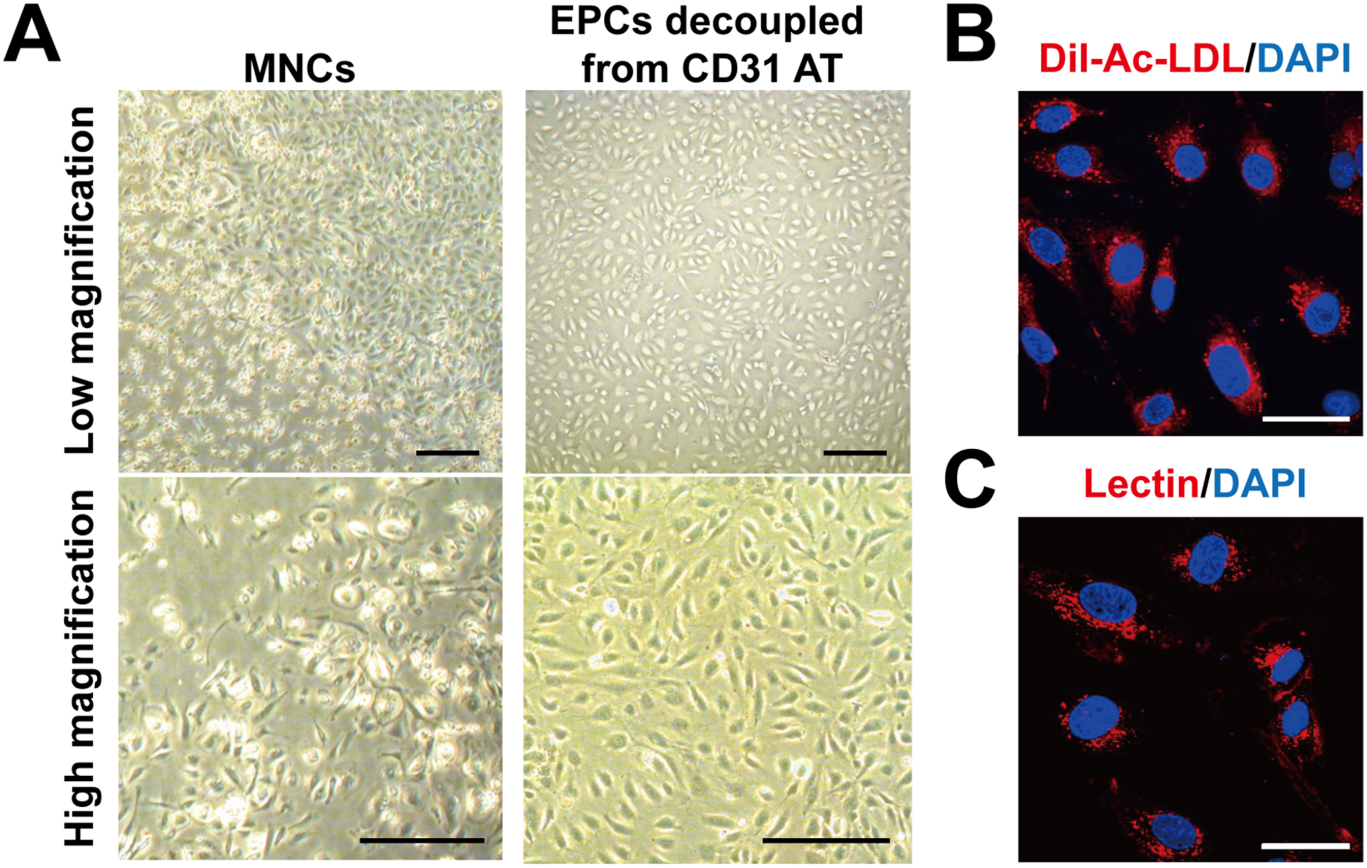
Preparation of foreign material-free EPCs from human cord blood MNC culture by magnetic cell sorting isolation with CD31 aptamer and decoupling processes. (A) Two-week culture of cord blood MNCs were subjected to magnetic bead isolation process with CD31 aptamers, followed by decoupling from CD31 aptamers with decoupling buffer. Bright field images (high and low magnification) of the MNCs and the EPCs decoupled from CD31 aptamers are shown. Scale bar = 100 μm. (B, C) Endothelial characteristics of the EPCs decoupled from CD31 aptamers were determined by staining with DiI-Ac-LDL (B) or lectin (Ulex europaeus agglutinin I) (C). Fluorescence images were taken by confocal microscope (Olympus FluoView FV1000). Scale bar = 40 μm. Representative data from three independent experiments are shown.

EPCs were established from the culture of MNCs from cord blood for more than three weeks [12]. In two-week culture of cord blood MNCs, heterogeneous morphology of cells (Fig 5A) and existence of CD31-negative cells along with CD31-positive cells were observed (data not shown). To expedite the establishment of EPCs in culture, we isolated CD31-positive cells from two-week culture of cord blood MNCs with biotin-labled CD31 aptamers and streptavidin magnetic beads and decoupled CD31 aptamers to generate foreign material-free EPCs as described in S5 Fig. CD31 aptamer-isolated and decoupled cells from cord blood MNC culture generated homogeneous cells in the subsequent culture (Fig 5A). Flow cytometry analysis showed CD31 aptamer-isolated and decoupled cells were positive for EPC surface markers such as CD31 (99.7%), KDR (43.3%), and VE-cadherin (92.8%) (S6 Fig). CD31 aptamer-isolated and decoupled cells in the culture showed endothelial characteristics such as uptake of Ac-LDL and staining by lectin (Fig 5B and 5C). These results demonstrate that foreign material-free EPCs can be isolated from heterogeneous mixture of cells using CD31 aptamers.

### Neovascularization by the CD31 aptamer-isolated and decoupled EPCs

**Fig 6:**
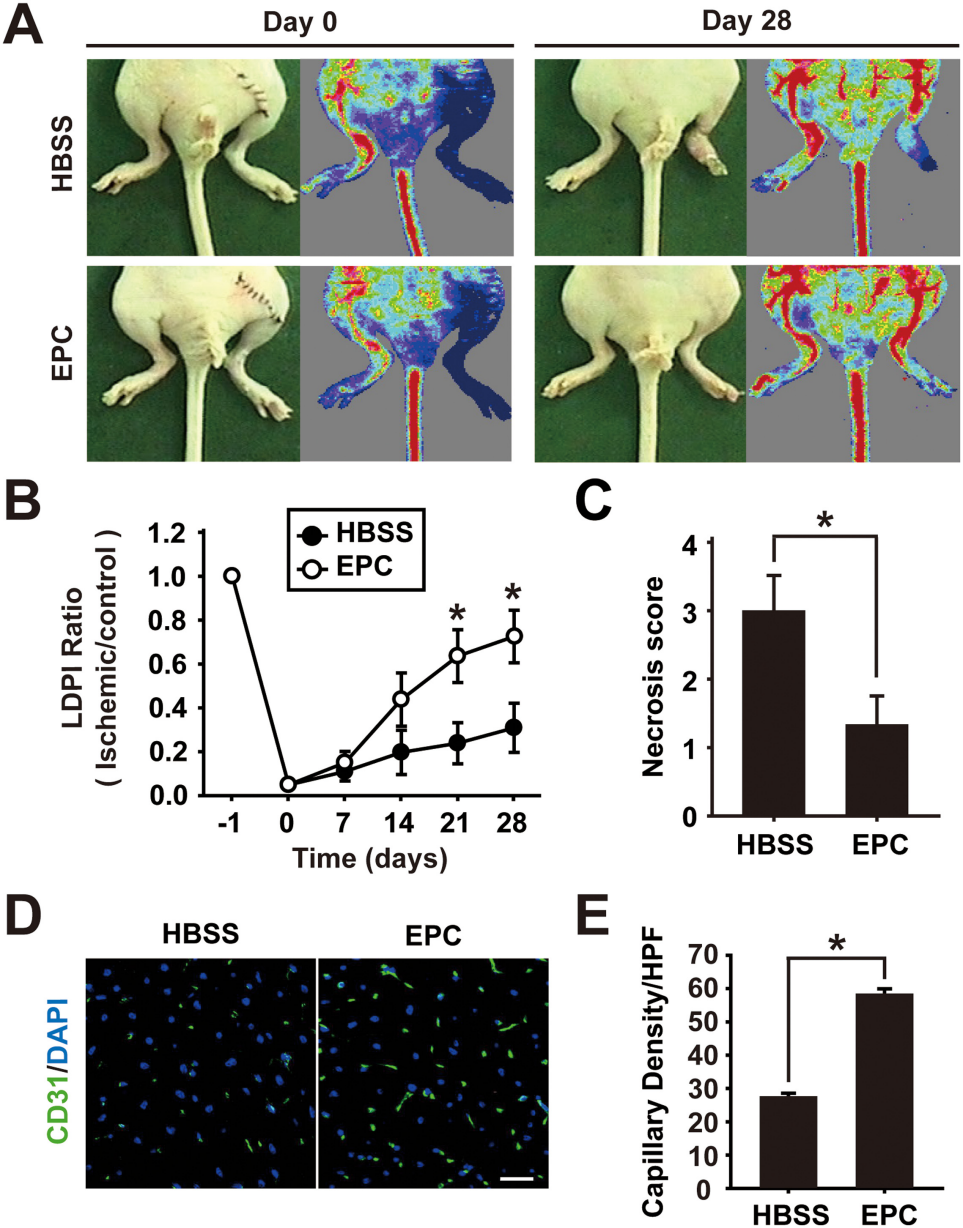
Foreign material-free EPCs enhance restoration of blood flow and limb salvage from ischemic injury. (A) The foreign material-free EPCs were injected intramuscularly to injury site of nude mice with hindlimb ischemia (1×10^6^ cells/injection). Representative images of mice with laser Doppler perfusion imaging (LDPI) on day 0 and day 28 after injection with HBSS buffer (n = 6) or EPCs (n = 6) are shown. (B) Time-course quantitative analysis of blood flow by LDPI of hindlimb ischemia-induced mice injected with EPCs or HBSS is shown. (C) Statistical analysis of necrosis score on day 28 is shown. (D) Immunostaining of CD31-positive capillaries (green) in salvaged hindlimbs on day 28 after intramuscular injection of HBSS or EPCs is shown. (E) Quantitative analysis of capillary density in salvaged limbs on day 28 by counting the number of CD31-positive capillaries per high-power field (HPF) is shown. * indicates p < 0.05 vs HBSS by Student’s t-test (n = 6 for EPC and n = 6 for HBSS respectively).

CD31-postive cells isolated from peripheral blood or bone marrow were previously shown to contribute to the repair of ischemic vascular injuries [21, 22]. Therefore, we tested whether the foreign material-free CD31-positive cells decoupled from CD31 aptamer-conjugated microbeads could contribute to the vascular repair from ischemic injuries. Femoral artery of nude mice was ligated surgically to introduce ischemic injuries and the foreign material-free CD31-positive cells were injected intramuscularly to the site of ischemic injury on the following day (1 x 10^6^ cells/injection). Mice were monitored up to day 28 after EPC injection with time-course measuring of blood flow. Analysis of mice on day 28 showed that EPC injection significantly improved blood flow and limb salvage compared with control HBSS injection (Fig 6A, 6B and 6C). Immunohistochemistry analysis of salvaged limbs showed that EPC injection increased CD31-positive capillaries compared with control HBSS injection (Fig 6D and 6E). These results demonstrate that EPCs established from heterogeneous culture of cord blood MNCs using CD31 aptamer isolation and decoupling protocol are functional and contribute to the recovery from ischemic injuries.

## Discussion

Reliable targeting of cell surface molecules is an important task in isolation, visualization, or delivery of drugs to the cells of interest. Thus far antibodies are the most widely used targeting reagent with high affinity and specificity in biomedical applications [35]. Besides high binding affinity and specificity comparable to those of antibodies, aptamers provide advantages over antibodies, including convenient synthesis and modifications, acceptable immunogenicity, and rapid tissue penetration [36]. The short production time and the straightforward modification and functionalization of aptamers make them suitable for attachment of functional groups in order to visualize or direct drugs into the targeting cells [3]. Isolation of cells with a low immunogenic reagent is favorable in transplanting cells into patients for therapeutic purposes [37, 38]. The rapid tissue penetration is beneficial in visualizing the target cells *in vivo* [1]. However, application of aptamers to cells requires further adjustments as aptamers were selected for the interaction with the purified proteins *in vitro* [39, 40]. In this study, we tested the aptamers selected *in vitro* using the purified extracellular part of CD31 protein for application to cells and established the protocols for detection, visualization, and isolation of EPCs, which provide an advantage in preparation of cells for therapeutic transplantation.

We tested three clones of CD31 aptamer on EPCs and 293FT cells. Although all three clones of the CD31 aptamer showed interaction with EPCs, they also showed non-specific interaction with 293FT cells (Fig 2 and S2 Fig). Our aptamers are Slow Off-rate Modified Aptamers (SOMAmers), which, like many nucleic acid drugs, may show non-specific and low-affinity interaction with various proteins in the μM range [41, 42]. This suggests that aptamers should be in the nM or better affinity range in order to show target-specific interaction. Our CD31 aptamer clones have Kd values in the nM range (AT-1, 2.28 nM; AT-2, 3.36 nM; AT-3, 1.14 nM). To resolve non-specific interaction with non-cognate targets, we applied equilibrium and kinetic challenges by addition of dextran sulfate as a polyanionic competitor and washing repeatedly, as previously reported [31, 32, 36]. In our fine tuning, 0.2 mM dextran sulfate eliminated non-cognate interaction but did not diminish specific interaction. However, high concentrations of dextran sulfate (1–2 mM) disrupted specific interaction with EPCs. In the case of EGFR aptamers, dextran sulfate was not necessary to show the specific interaction with cells but 1 mM dextran sulfate was adopted in the purification of EGFR protein (data not shown) [31]. These results suggest that individual optimization of aptamers in each application may be necessary in order to reveal specific interaction over non-specific interaction with non-cognate targets.

The advantage of CD31 aptamers over CD31 antibodies is decoupling of aptamer from EPCs after isolation (Figs 4D and 5). Efficiency of CD31 aptamers was as good as that of CD31 antibodies in isolating EPCs (Fig 4B). In transplanting EPCs for therapeutic purposes, the absence of foreign material is preferred [15]. Isolation of EPCs using antibodies leaves a cause for concern regarding transplanting EPCs with antibodies and magnetic beads attached to the surface. In the development of therapeutic antibodies, the preference has shifted from mouse monoclonal antibodies to chimeric antibodies, humanized antibodies and finally to human antibodies in an effort to minimize the safety issue and diminished efficacy caused by immunogenicity of antibodies [43]. Even with human antibodies, immunogenicity concerns remain due to diverse genotypes, different immunoglobulin G allotypes, and generation of anti-idiotypic antibodies [26, 44]. Magnetic beads, when used in isolation of endothelial cells, were shown to have a late detrimental effect on cell proliferation and metabolism [27]. Furthermore, aptamers and oligonucleotides could activate Toll-like receptor pathway or generate antibodies against them due to the presence of CpG motif [45, 46]. We developed a simple and universal protocol for release of aptamers and magnetic beads from EPCs by a short-time treatment with a high concentration of dextran sulfate after isolation of EPCs. In comparison, a common-decoupling competitor of antibody-antigen interaction has not been reported [32, 47]. Our simple protocol may provide a universal platform for isolation of foreign material-free target cells, which is expected to enhance the efficacy in therapeutic cell transplantation.

## Conclusion

In conclusion, we generated CD31 aptamers that specifically recognize human CD31 on the cell surface and established a protocol for detection and visualization in flow cytometry and fluorescence microscopy. Furthermore, we established a protocol for isolating foreign material-free EPCs from heterogeneous mixture of cells using CD31 aptamers and magnetic beads. We expect our results contribute to the expedition of recovery from ischemic conditions and clinical applications of stem cells.

## Supporting Information

S1 Fig CD31 is expressed on EPCs but not on 293FT cells. Flow cytometry analysis of EPCs (A) or 293FT (B) cells with FITC-labeled CD31 antibodies and isotype control antibodies is shown (n = 5).
(TIF)

S2 Fig Dextran sulfate resolves non-specific interaction of CD31 aptamers. The mixture of EPCs and 293FT cells were incubated with CD31 aptamers (AT-2 and AT-3, Cy5-labeled) and various concentrations of dextran sulfate. Histogram from flow cytometry analysis is shown (n = 3).
(TIF)

S3 Fig No effects of dextran sulfate on cell viability. (A) Flow cytometry analysis with 7-AAD staining after incubation of EPCs or 293FT cells with various concentrations (0, 0.2, 0.5, 1, and 2 mM) of dextran sulfate at room temperature for 15 minutes (n = 5). (B) EPCs or 293FT cells were incubated with various concentrations (0, 0.2, 0.5, 1, and 2 mM) of dextran sulfate at room temperature for 30 minutes and subjected to MTT assay (n = 4).
(TIF)

S4 Fig Interaction of CD31 aptamers with human CD31 but not with mouse CD31. (A) Mouse ESCs were differentiated into EBs and day 6 mouse EB-derived cells were subjected to flow cytometry analysis with control isotype antibodies (left panels) or control scrambled EGFR-FTIC aptamers (right panel). (B) Day 6 mouse EB-derived cells were subjected to flow cytometry analysis with CD31 aptamers (AT-1, Cy5-labeled) in combination with FITC-labeled anti-human CD31 antibodies (upper panels) or PE-labeled anti-mouse CD31 antibodies (lower panels) (n = 3).
(TIF)

S5 Fig Schematic description of EPC isolation with CD31 aptamers and decoupling from CD31 aptamers is shown. (TIF)

S6 Fig Maintenance of EPC surface markers in foreign material-free EPCs. Flow cytometry analysis of foreign material-free EPCs isolated from two-week cord blood MNC culture using CD31 aptamers and decoupling protocol is shown (n = 4).
(TIF)

S1 Table Aptamer sequences. 5-(N-naphthylcarboxyamide)-2’-deoxyuridine (NapdU) aptmaers are shown. 6: dTTPs → dUTPs.
(TIF)
