# Supplementary figures and images for: Isolation of Foreign Material-Free Endothelial Progenitor Cells Using CD31 Aptamer and Therapeutic Application for Ischemic Injury
Source: PLoS One. 2015 Jul 6;10(7):e0131785. doi: 10.1371/journal.pone.0131785 (PMC4493074; doi:10.1371/journal.pone.0131785)

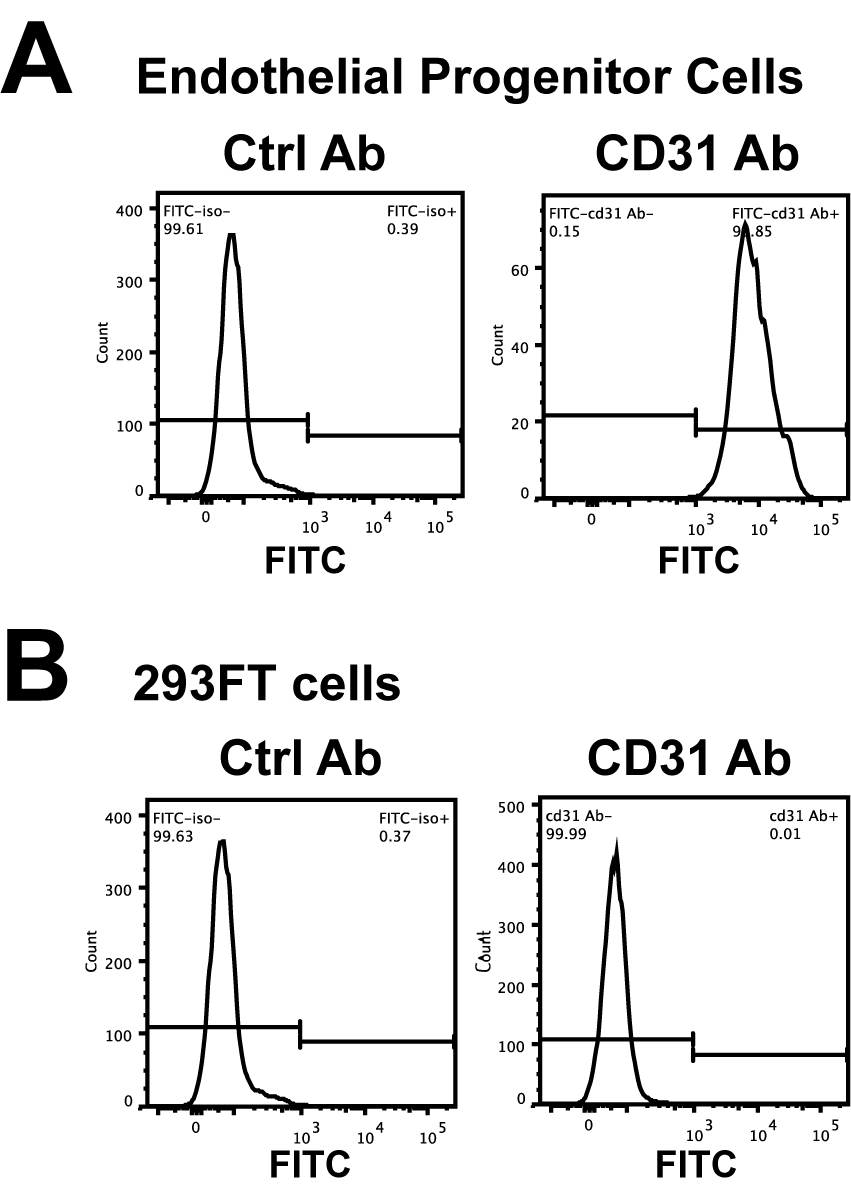

Supplement: S1 Fig — Flow cytometry analysis of EPCs (A) or 293FT (B) cells with FITC-labeled CD31 antibodies and isotype control antibodies is shown (n = 5). (TIF) [file pone.0131785.s001.tif]

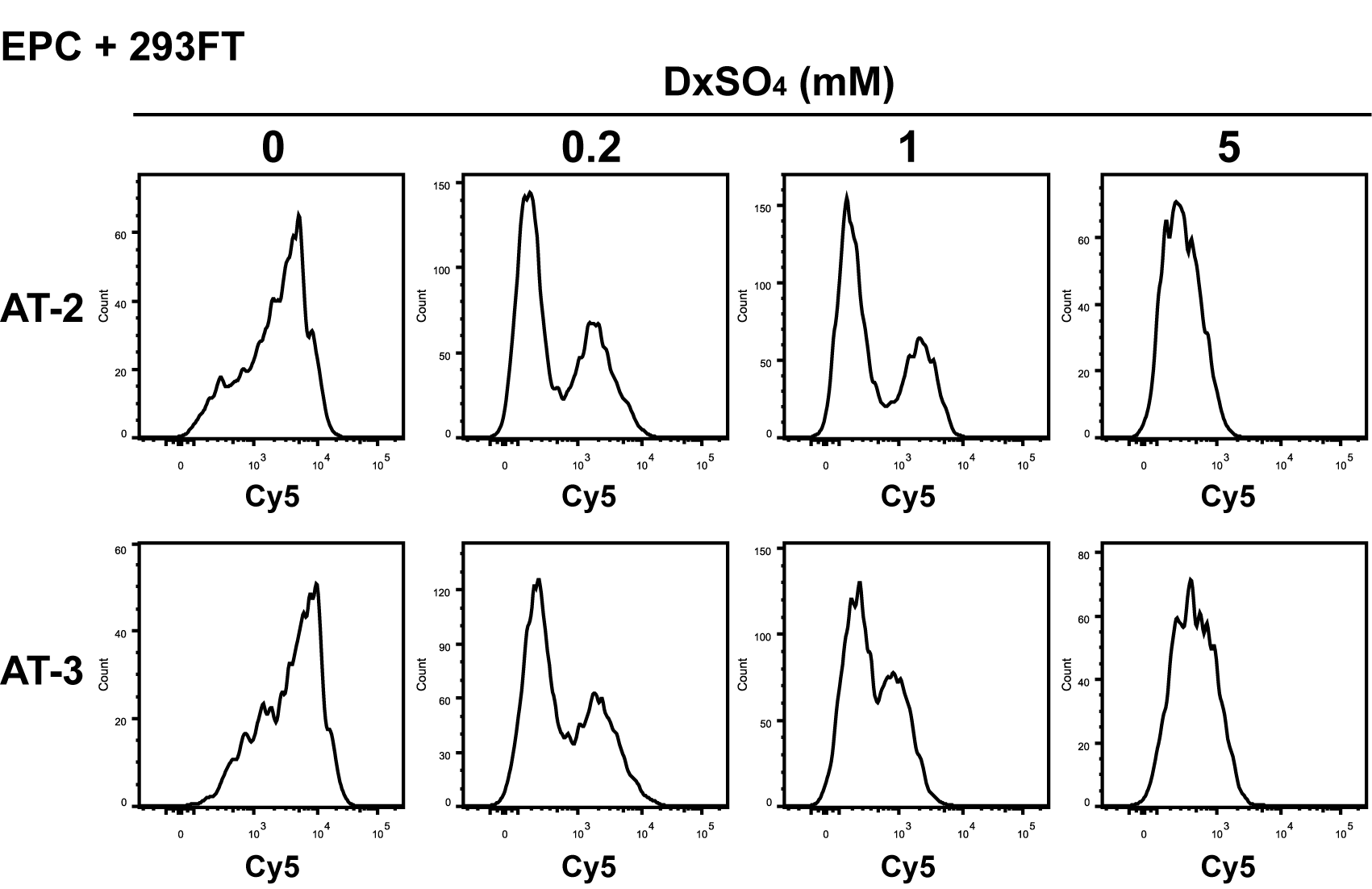

Supplement: S2 Fig — The mixture of EPCs and 293FT cells were incubated with CD31 aptamers (AT-2 and AT-3, Cy5-labeled) and various concentrations of dextran sulfate. Histogram from flow cytometry analysis is shown (n = 3). (TIF) [file pone.0131785.s002.tif]

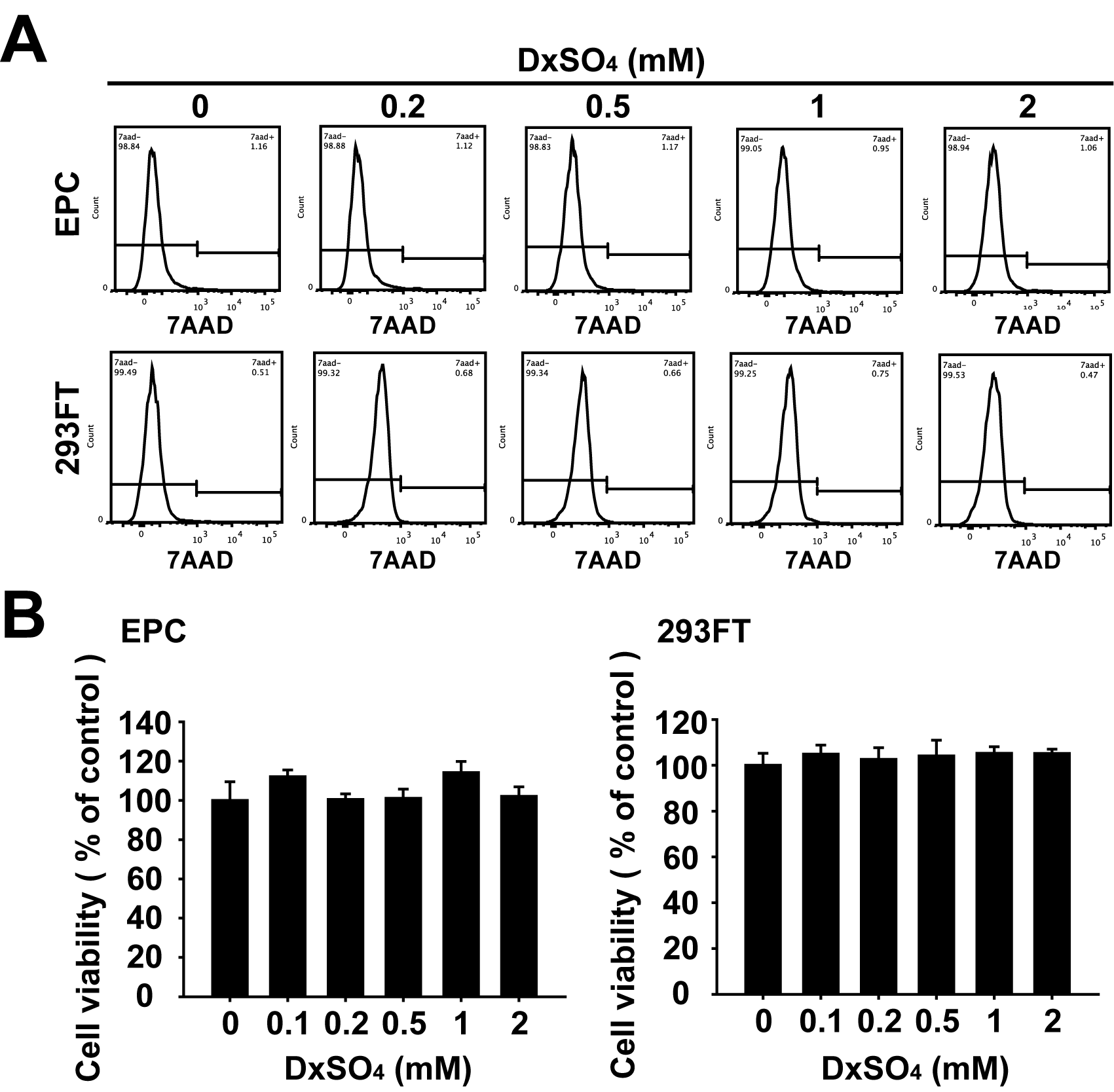

Supplement: S3 Fig — (A) Flow cytometry analysis with 7-AAD staining after incubation of EPCs or 293FT cells with various concentrations (0, 0.2, 0.5, 1, and 2 mM) of dextran sulfate at room temperature for 15 minutes (n = 5). (B) EPCs or 293FT cells were incubated with various concentrations (0, 0.2, 0.5, 1, and 2 mM) of dextran sulfate at room temperature for 30 minutes and subjected to MTT assay (n = 4). (TIF) [file pone.0131785.s003.tif]

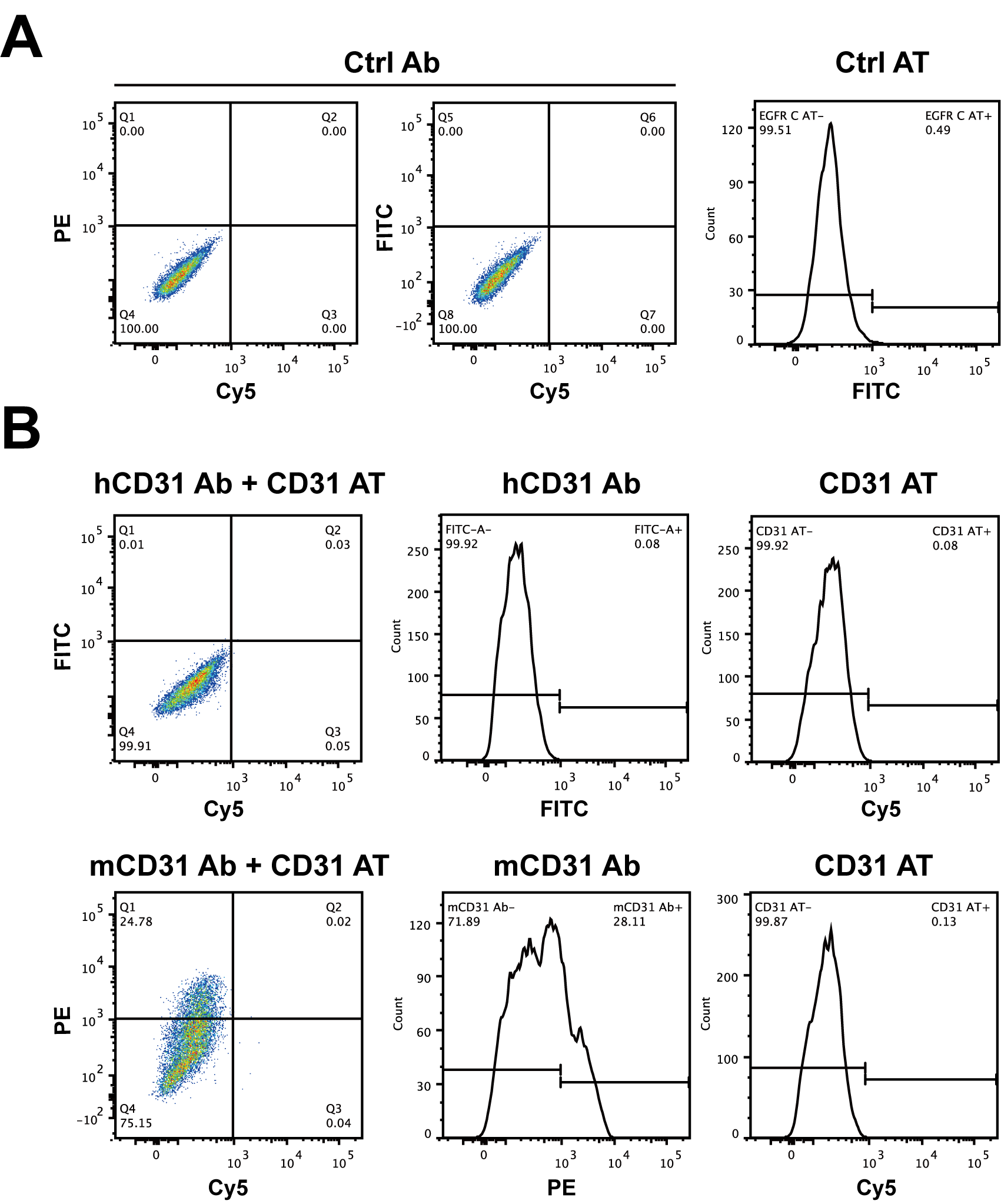

Supplement: S4 Fig — (A) Mouse ESCs were differentiated into EBs and day 6 mouse EB-derived cells were subjected to flow cytometry analysis with control isotype antibodies (left panels) or control scrambled EGFR-FTIC aptamers (right panel). (B) Day 6 mouse EB-derived cells were subjected to flow cytometry analysis with CD31 aptamers (AT-1, Cy5-labeled) in combination with FITC-labeled anti-human CD31 antibodies (upper panels) or PE-labeled anti-mouse CD31 antibodies (lower panels) (n = 3). (TIF) [file pone.0131785.s004.tif]

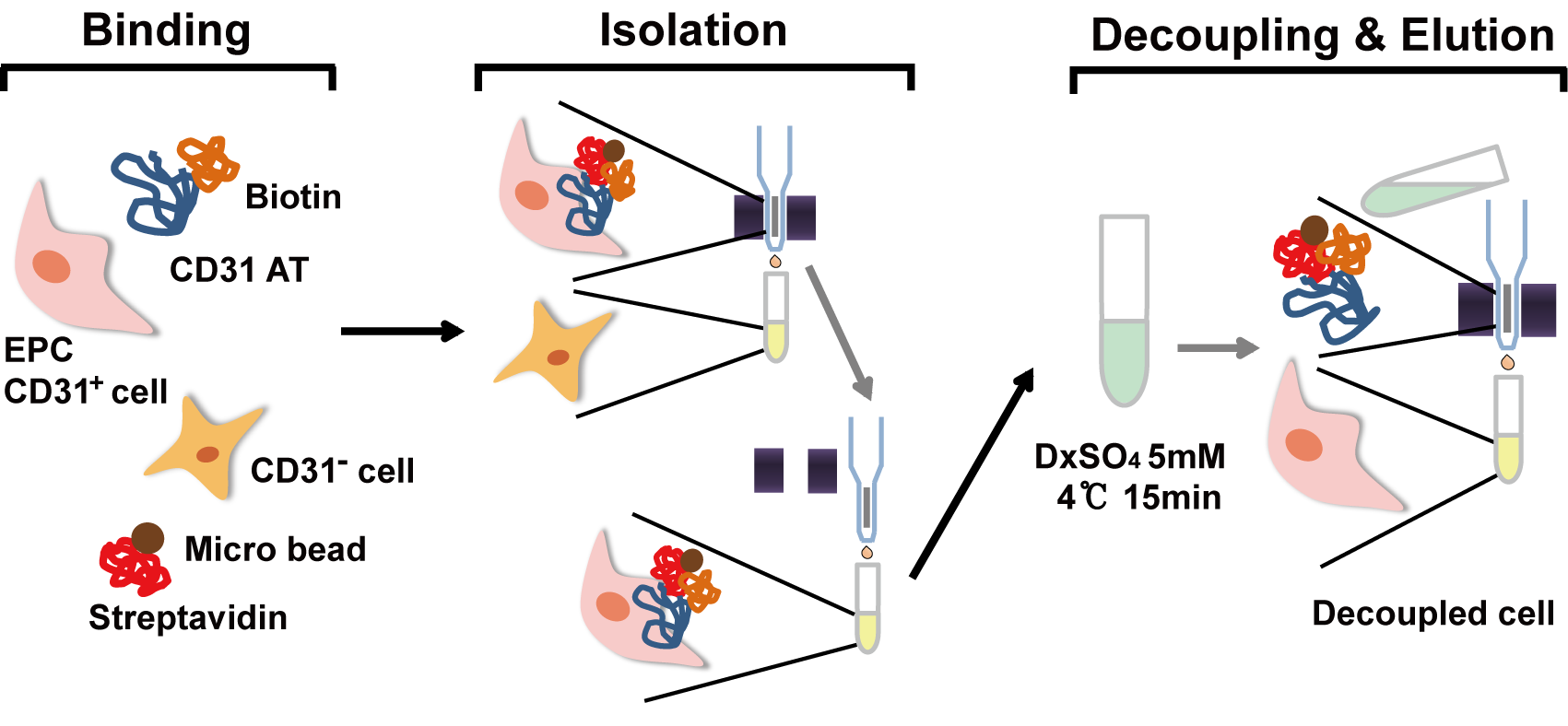

Supplement: S5 Fig — (TIF) [file pone.0131785.s005.tif]

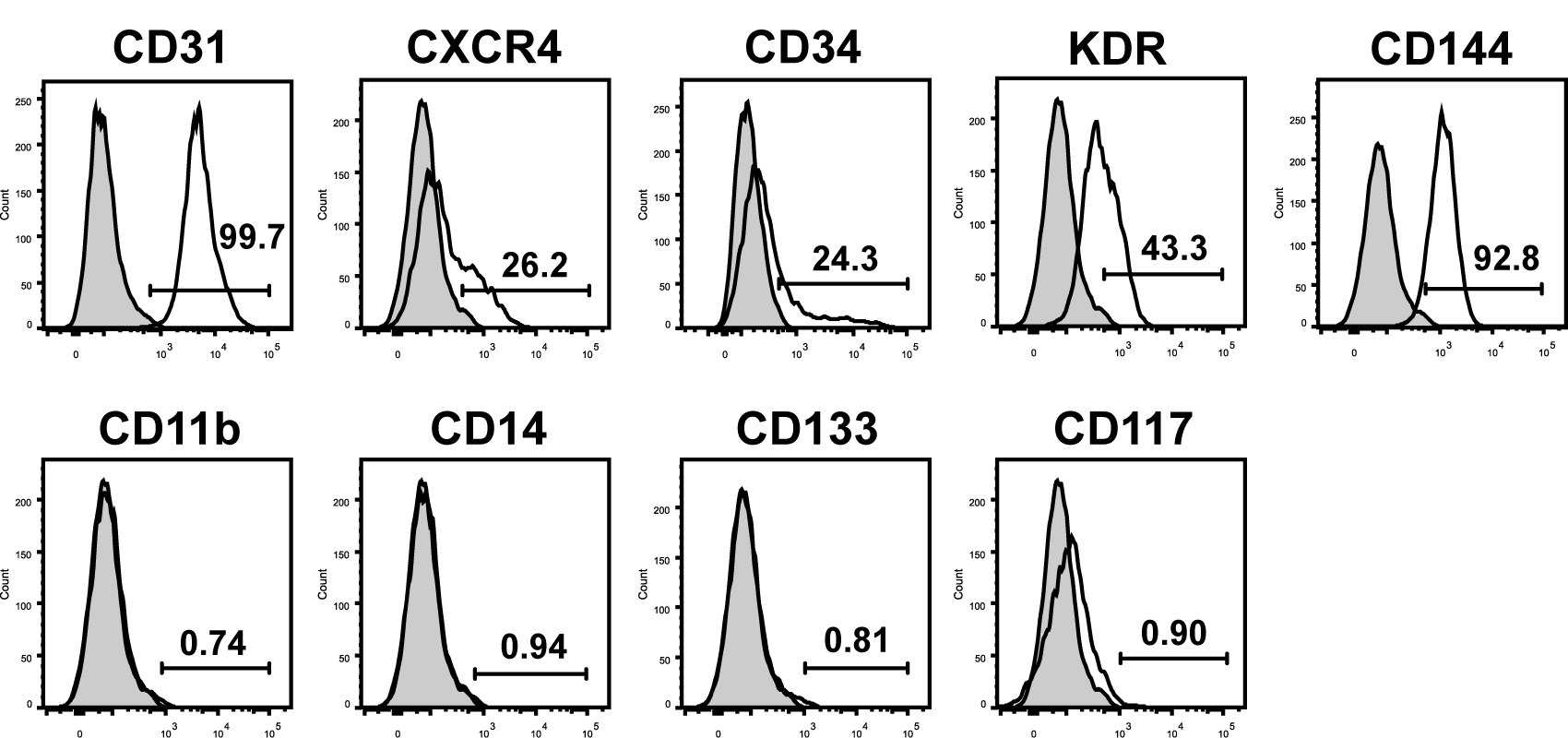

Supplement: S6 Fig — Flow cytometry analysis of foreign material-free EPCs isolated from two-week cord blood MNC culture using CD31 aptamers and decoupling protocol is shown (n = 4). (TIF) [file pone.0131785.s006.tif]

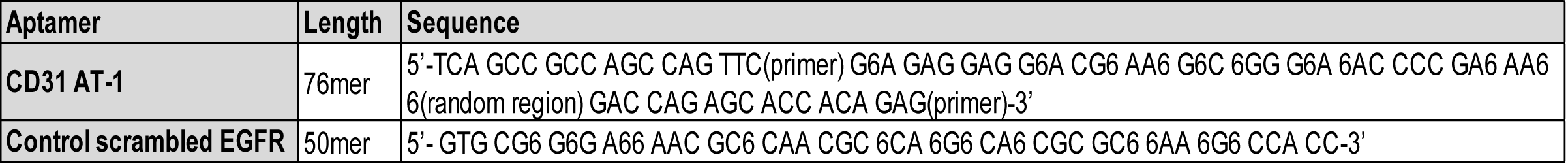

Supplement: S1 Table — 5-(N-naphthylcarboxyamide)-2’-deoxyuridine (NapdU) aptmaers are shown. 6: dTTPs → dUTPs. (TIF) [file pone.0131785.s007.tif]
